# Effect of saline irrigation on *Tetragonia tetragonioides* (Pall.) Kuntze grown on different soil types under greenhouse conditions

**DOI:** 10.1002/jsfa.70308

**Published:** 2025-11-12

**Authors:** Giulia Atzori, Michele Petrillo, Giulia Mozzo, Simone Bernardi, Stefania Caparrotta, Nadia Bazihizina, Stefano Mancuso, Elisa Masi

**Affiliations:** ^1^ Institute of Sustainable Plant Protection National Research Council of Italy (CNR‐IPSP) Florence Italy; ^2^ Department of Agriculture, Food, Environment and Forestry (DAGRI) University of Florence Florence Italy; ^3^ CEABN/InBIO, Centre for Applied Ecology'Prof. Baeta Neves', Institute of Agronomy University of Lisbon Lisbon Portugal; ^4^ Department of Biology Università degli Studi di Firenze Florence Italy

**Keywords:** saline agriculture, New Zealand spinach, halophyte, salt‐tolerant crop, phytodesalination

## Abstract

**BACKGROUND:**

Climate change and population growth are major challenges for sustainable food production, particularly in regions affected by water scarcity and soil salinization. In this context, halophytes represent promising candidates as alternative and sustainable food crops for salt‐affected areas.

**RESULTS:**

The results of an experiment evaluating the salt responses and nutritional values of the edible halophyte *Tetragonia tetragonioides* grown at three salinity levels (0, 100, and 200 mmol L^−1^ NaCl) in two soils with different textures revealed similar growth parameters under control and 100 mmol L^−1^ NaCl conditions in both soils. Conversely, the 200 mmol L^−1^ NaCl treatment negatively affected plant growth, with plants grown in Soil A + S consistently outperforming those grown in Soil A. At moderate salinity (100 mmol L^−1^ NaCl), water productivity increased, driven by lower stomatal conductance and transpiration rates. Moreover, mineral analysis of edible leaves revealed a progressive accumulation of Na and Cl with increasing salinity, especially in Soil A‐grown plants. On the other hand, salt‐grown plants had higher leaf Cu, Zn and Mn concentrations, key micronutrients often lacking in human diets. Furthermore, soils sampled near plant roots showed lower electrical conductivity (EC) compared to distant soil samples, confirming a phytodesalination effect by *T. tetragonioides*.

**CONCLUSION:**

The obtained results indicate that *T. tetragonioides* can be successfully cultivated in moderately saline soils (EC < 20 dS m^−1^), providing dual benefits: production of mineral‐enriched edible biomass and partial remediation of salt‐affected soils. This dual role highlights its potential as a valuable crop for sustainable agriculture in marginal saline environments. © 2025 The Author(s). *Journal of the Science of Food and Agriculture* published by John Wiley & Sons Ltd on behalf of Society of Chemical Industry.

## INTRODUCTION

Salinity limits agricultural productivity globally, particularly in arid and semi‐arid regions where soil salinization is prevalent.^1^ The surface area affected by salinization is increasing up to 1.5 Mha each year due to this phenomenon.^2^ The FAO's Global Status of Salt‐Affected Soils report^1^ provides the latest estimate on the areas of salt‐affected soils in the world, reaching a total area of salt‐affected soils of 1381 Mha, or 10.7% of the total global land area. Climate change exacerbates this problem, particularly in coastal regions, where rising sea levels, caused mainly by the melting of land ice and the thermal expansion of the oceans due to global warming, affects soil salinization through the intrusion of saline water in aquifers and soils.^1^ Drought events further increase the issue of secondary soil salinization, negatively affecting agricultural productivity.^3^

The accumulation of salts in soils can severely affect plant growth, leading to reduced crop yields and compromising food security. Excess salts in the soil hinder root water uptake, leading to wilting and reduced growth. Furthermore, high concentrations of sodium and chlorides can be toxic to plants, interfering with essential physiological processes and causing nutritional imbalances.^4^ Since the work conducted by Maas and Hoffman,^5^ salt tolerance has generally been defined based on a threshold salinity measured using the soil's electrical conductivity (EC, generally measured as ECe – i.e. of the saturated paste; or EC1:5 – 1:5 soil water solution). Soil EC is a measure of soil salinity and is a critical parameter that reflects the salinity levels in the root zone, influencing plant growth and development.^6^, ^7^ However, EC values alone do not fully explain the plant's response to salinity, as these values must be interpreted in the context of pedoclimatic conditions, including soil type and climatic factors such as temperature and humidity.^4^, ^8^ Indeed, these conditions alter plants’ ability to absorb water and nutrients, thereby affecting their overall growth and productivity.^8^

Previous studies^9^, ^10^, ^11^ have shown that soil type, whether sandy or clayish, can have a substantial impact on the salinity tolerance of plants. Sandy soils, with their larger particle sizes and better drainage, often exhibit different salinity dynamics compared to clay soils, which have higher water retention capacity but may also retain more salts.^12^ Moreover, climatic factors such as temperature and seasonality play a crucial role in plant physiology and growth. For instance, higher temperatures increase evapotranspiration rates, leading to higher salt accumulation in the rhizosphere.^13^ As a result, the interactions between soil properties and climatic conditions can substantially modify plants' salinity tolerance thresholds.^14^

In addition to specific climatic and soil conditions, the intrinsic salt tolerance of the crops is also a key factor in determining productivity in saline environments. Among potential crops for saline soils, halophytes are defined as salt‐loving plants that can complete their life cycle with salt concentrations in the root zone of 200 mmol L^−1^ NaCl.^15^ Out of an estimated 365 000 higher plant species, around 625 species are classified as halophytes, representing just 0.2% of the plant species.^16^ Despite their promising nutritional and agronomic potential for salt‐affected areas, many halophytes remain underutilized.^1^, ^17^, ^18^

Among these, *Tetragonia tetragonioides* (Pall.) Kuntze, commonly known as New Zealand spinach and hereafter referred to as Tetragonia, has garnered attention for its combination of high salt tolerance and nutritive value investigated in hydroponics.^19^, ^20^, ^21^, ^22^, ^23^ Understanding how pedoclimatic factors interact with soil salinity is essential for optimizing Tetragonia cultivation in saline environments. Indeed, previous studies have shown fundamental differences in salinity responses in glycophytes between soil and solution culture^24^ highlighting the need to validate the assumption that responses in hydroponics mimic those in soils. Thus, building on our recent findings that Tetragonia seedlings tolerate salinity levels up to 200 mmol L^−1^ NaCl in a rhizoslide system with filter paper and in hydroponics, with optimal growth at 100 mmol L^−1^,^19^, ^22^ this study investigates the impact of salinity and soil type on the growth and productivity of Tetragonia. We test the hypothesis that, as also observed in glycophytes, salt tolerance of this halophyte is affected by soil texture, with an improved performance in the soil with a greater percentage of sand due to reduced salt accumulation. In particular, we investigate how soil texture affects soil EC values and, consequently, plant growth and ion accumulation. Furthermore, by analysing ion accumulation in plant tissues, we evaluate whether soil texture and salinity affect the nutritive value of this crop, providing insights into the physiological mechanisms underlying its adaptation to high root zone salinity.

## MATERIALS AND METHODS

### Growth conditions and experimental design

A pot trial was carried out in 2023 at the greenhouse facilities of the Department of Agriculture, Food, Environment and Forestry (DAGRI) of the University of Florence, Italy (43° 48′ 58.6″ N, 11° 11′ 58.1″ E). Temperature trends were recorded with a temperature data logger during the experiment: mean temperature, 23.5 °C; maximum temperature, 42.5 °C; minimum temperature, 12.5 °C.

Seeds of *Tetragonia tetragonioides* were obtained from the Tuttosemi Company (www.tuttosemi.com) and germinated in a dark chamber at 18.5 °C 2 months prior to the start of the experiment. After germination, young plantlets were transplanted into 3.5 L pots (diameter 25 cm) filled with two different soils, i.e. Soil A and Soil A + S. Soil A was collected in Radda in Chianti (Siena) (43° 30′ 14.2″ N, 11° 19′ 56.2″ E) from an agricultural site, while Soil A + S was obtained by mixing 50% Soil A and 50% sand. The chemical and physical characteristics of the soils used are reported in Table 1.

**Table 1:** Physical and chemical characteristics of the two soils used in the experiment

Soil treatment	Sand	Clay	Loam	CEC	pH	EC1:5	N	C	Na	Ca	K	Mg
	(%)	(%)	(%)	(meq 100 g^−1^)		(dS m^−1^)	(%)	(%)	(ppm)	(ppm)	(ppm)	(ppm)
A	50	30	20	12.80192	8.1	1.03	0.05	0.52	9.01	81.5	106.8	624.7
A + S	70	20	10	10.33685	8	0.77	0.04	0.5	7.02	40.8	51.3	302

Salinity treatments were supplied via irrigation using a half‐strength Hoagland nutrient solution supplemented with different NaCl concentrations: control (i.e., 0 mmol L^−1^ NaCl, EC ≈ 2 dS m^−1^), 100 mmol L^−1^ NaCl (EC ≈ 10 dS m^−1^) and 200 mmol L^−1^ NaCl (EC ≈ 20 dS m^−1^). The three salinity levels were used for both soils, for a total of six treatments corresponding to two soil conditions (Soil A and Soil A + S) and three salinity levels, each with nine biological replicates in a completely randomized block design. The experimental period lasted 4 weeks after treatment initiation, covering a complete crop cycle, with an intermediate harvest after 2 weeks (Hi; *n* = 3) and the final harvest (Hf; *n* = 6) after 4 weeks. During Hf, leaf material was collected and dried for further analysis of mineral element concentration or frozen for further analysis of chloride concentration.

### Soil characterization

Soil A and Soil A + S were analysed for their chemical and physical characteristics before the start of the experiment, as reported in Table 1.

Soil samples were analysed as follows. The cation exchange capacity (CEC) was determined with barium chloride and triethanolamine. An amount of 2 g soil sample was added to 25 mL barium chloride solution (BaCl_2_.2H_2_O) at pH 8.2. After centrifugation, the clear solution was used to determine total acidity. The sample was washed with 30 mL H_2_O, and after removing the supernatant the mass of the tube + sample was measured again. An amount of 25 mL magnesium sulfate solution (5 cmol L^−1^) was added to the tube with the sample. The tube was closed, shaken and then centrifuged. An amount of 10 mL of the clear solution was transferred to a flask containing 100 mL H_2_O, 10 mL of the buffer solution at pH 10 and the tip of an indicator spatula. Ethylenediaminetetraacetic acid solution was used to titrate the sample solution and the blank test solution to a light‐blue colour.

Soil N and C concentrations were determined from soil extracts according to the Dumas method, using an elemental analyser (Flash EA 1112, Thermo Fisher Scientific, Waltham, MA, USA).^25^, ^26^ Briefly, soil Ca, K, Mg and Na concentrations were extracted using an ammonium acetate solution at pH 7. After extraction, diluted samples were used to determine the concentrations of the ions utilizing inductively coupled plasma optical emission spectrometry (ICP OES) according to the method described in Guidi Nissim *et al*.^27^

To evaluate how salinity affected soil EC and pH, soil samples were collected from the central part of the pot after 2 (Hi) and 4 (Hf) weeks treatment. At Hf, additional soil samples were collected in proximity to the root collar (~3 cm from the centre of the pot) and to the side of the pot to examine the potential spatial difference in soil EC linked to plant salt uptake activity. After drying the soils at 90 °C, soil EC and pH were measured from a 1:5 soil–water suspension and its pH and EC measured, respectively, with a pH meter (MeterLab, Radiometer Copenhagen, Denmark) and an EC meter (Conductimeter GLP 31, Crison Instruments, Barcelona, Spain). The soil EC1:5 values were then multiplied by a factor of 5 to obtained soil EC values.

### Plant growth and yield

Plant growth was monitored throughout the experiment by measuring the increase in leaf area cover, as Tetragonia is characterized by a prostrate growth. Each plant was photographed from above once a week together with a ruler, and all pictures were analysed using ImageJ to determine the leaf area cover.

Plants were harvested at both Hi (*n* = 3) and Hf (*n* = 6) to determine shoot fresh and dry weight. Leaves and stems were separated and their fresh weight was recorded. All samples were then oven‐dried at 70 °C until constant weight to determine their dry weights.

### Water use

Water use efficiency (WUE) and water productivity (WP) were calculated at Hf on six replicates per treatment. WUE was calculated as the ratio between the shoot oven‐dry biomass and total evapotranspiration (ET) throughout the crop cycle, as follows:

(1) $$\mathrm{WUE}=\text{Plant shoot}\;\mathrm{dry}\;\text{biomass}\;\left(g\right)/\mathrm{ET}\;\left(L\right)$$

WP was calculated as the ratio between the fresh marketable biomass (yield) and total ET throughout the crop cycle, as in the following formula:

(2) $$\mathrm{WP}=\text{Fresh marketable biomass}\;\left(g\right)/\mathrm{ET}\;\left(L\right)$$

The second equation was used to relate biomass production to ET more accurately, since the fresh shoot represents the edible portion of Tetragonia.

To estimate ET for all plants, pot weight was recorded before and after each irrigation event. In addition, for each treatment, one of the (*n* = 9) pots was placed on a scale connected to a datalogger that continuously recorded its weight at 10 s intervals.

### Leaf gas‐exchange parameters

Stomatal conductance (gsw) and the actual quantum efficiency of photosynthetic electron transport through photosystem 2 (ΦPSII) were measured using a steady‐state portable handheld porometer/fluorometer (LI‐600, LI‐COR Biosciences, Lincoln, NE, USA). The measurements were taken on healthy, newly fully expanded leaves on six plants per treatment on a weekly basis.

Leaf gas‐exchange parameters were also determined using the open gas‐exchange system Li‐6400 XT (LI‐COR) at Hi and Hf on six plants per treatment. Through the open gas‐exchange system, the net photosynthetic rate, stomatal conductance and intercellular CO_2_ concentrations were measured on the youngest fully expanded leaves using the following settings: ambient relative humidity, reference CO_2_ concentration of 400 μmol mol^−1^, flow rate of 500 μmol s^−1^, chamber temperature of 25 °C and photosynthetically active radiation of 2000 μmol m^−2^ s^−1^.

### Concentration of ions in the edible leaves

Oven‐dried leaf samples (six replicates of ~0.1 g each per treatment collected at Hf) were mineralized into Teflon vessels using a Mars Xpress microwave (CEM Corp., Matthews, NC, USA) with 10 mL HNO_3_. The microwave settings were as follows: power, 1600 W applied at 100%; ramp of 15:00 min to reach 200 °C; held for 15:00 min. At the end of this process, the final volume of the solution was obtained by adding 25 mL water 18 MΩ. Diluted extracts were analysed for Na, K, Cu, Mn and Zn concentration by ICP OES (Iris Intrepid II, Thermo Fisher Scientific) based on atomic emission spectroscopy.

A chloride assay kit (MAK023, Sigma‐Aldrich, St Louis, MO, USA) was used for Cl^−^ quantification (six replicates per treatment collected at Hf). In brief, 75 μL of reagent was added to 25 μL of sample (leaf sap extracted from fresh samples) in a 96‐multi‐well plate and incubated for 15 min at room temperature, after which the absorbance level was measured with a spectrophotometer (Infinite 200, Tecan, Männedorf, Switzerland) at 620 nm (*A*_620_). The sap was adequately diluted with ultrapure water to fit the chloride calibration curve prepared from six NaCl concentrations (0, 0.4, 0.8, 1.2, 1.6 and 2 mmol L^−1^).

### Statistical analyses

The experimental set‐up followed a complete randomized design to ensure uniform experimental conditions. Collected data were analysed with GraphPad Prism 10 for Windows (GraphPad Software Inc., Boston, MA, USA). According to the different datasets, one‐way or two‐way analysis of variance (ANOVA) was performed to assess significant differences among the treatments. In the following section, the statistical analyses conducted for all datasets are specified.

## RESULTS

### Soil salinization

Fig 1 shows the EC values of soil samples collected after two (i.e., Hi) and four weeks (i.e., Hf) from the start of the treatments. Data obtained from soil samples collected before the introduction of treatments (not reported in the figure) were similar to those of control pots for both soils.

**Figure 1:**
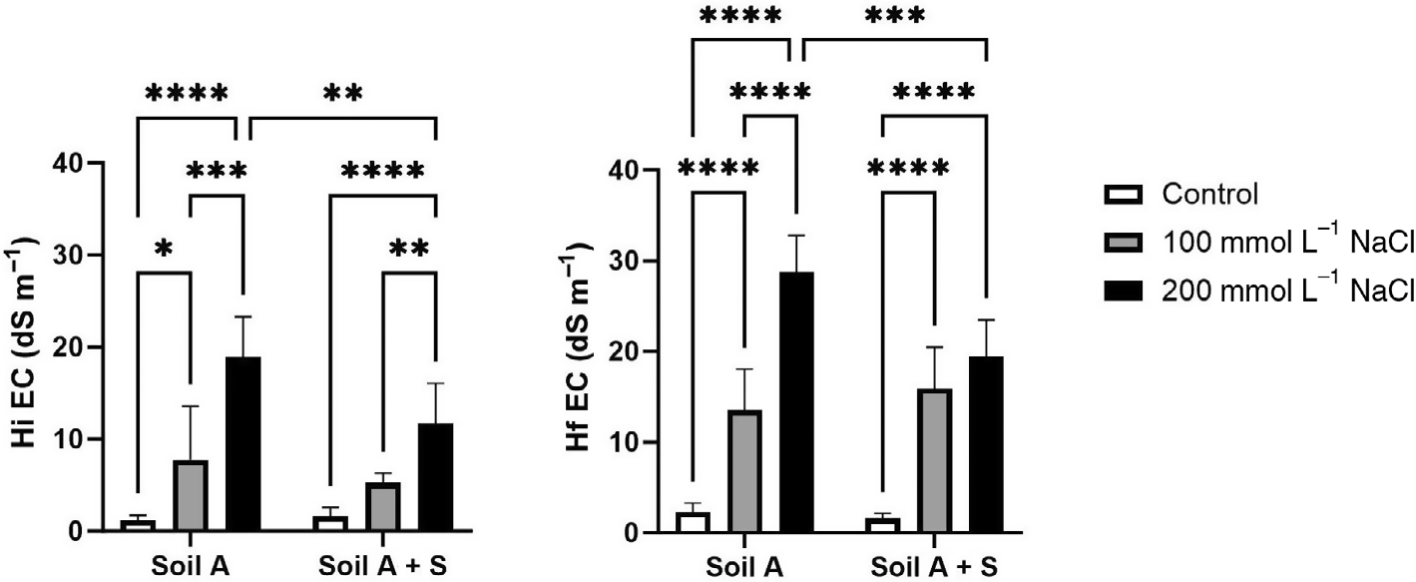
Salt accumulation in the soil after two (Hi) and four (Hf) weeks of treatment. Values are means (*n* = 6) ± standard deviation. Asterisks denote statistically significant differences at *P* < 0.05 (two‐way ANOVA and Tukey's test).

At both Hi and Hf, Soil A irrigated with 200 mmol L^−1^ NaCl had significantly higher EC compared to Soil A + S under the same treatment, reflecting the higher leaching capacity of Soil A + S compared to Soil A. This resulted in EC values ~10 dS m^−1^ higher than those in Soil A + S at both harvests. For instance, after two weeks of irrigation with 200 mmol L^−1^ NaCl, Soil A reached an average EC of ~20 dS m^−1^, whereas Soil A + S reached the same value two weeks later, at the end of the experiment.

At Hf, the potential spatial difference in soil EC linked with plant salt uptake activity was also examined by collecting soil samples near and far from the roots (Fig. 2).

**Figure 2:**
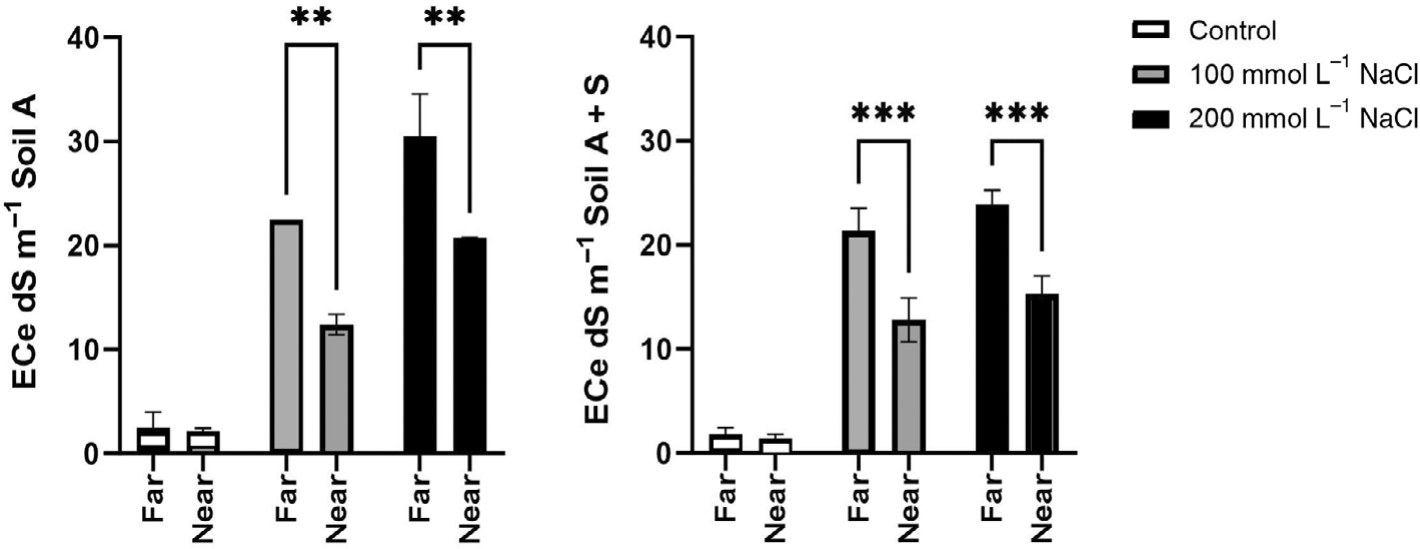
Soil EC at Hf according to proximity to root collar. ‘Near’ depicts values of soil collected at approx. 3 cm from the centre of the pot, while ‘Far’ depicts values of samples collected at the side of the pot. Values are means (*n* = 6) ± standard deviation. Asterisks denote statistically significant differences at *P* < 0.05 (two‐way ANOVA and Tukey's test).

For both soil texture and salt treatments, soil EC close to the root was significantly lower than that in the bulk soils (i.e., at the farthest point from the roots).

### Plant growth and yield

When comparing both shoot dry biomass and shoot fresh weight (i.e., yield), after 2 weeks of treatment (Hi) no significant differences emerged across salt and soil salinity treatments (data not shown). Fig 3 reports the shoot dry biomass and yield at the Hf.

**Figure 3:**
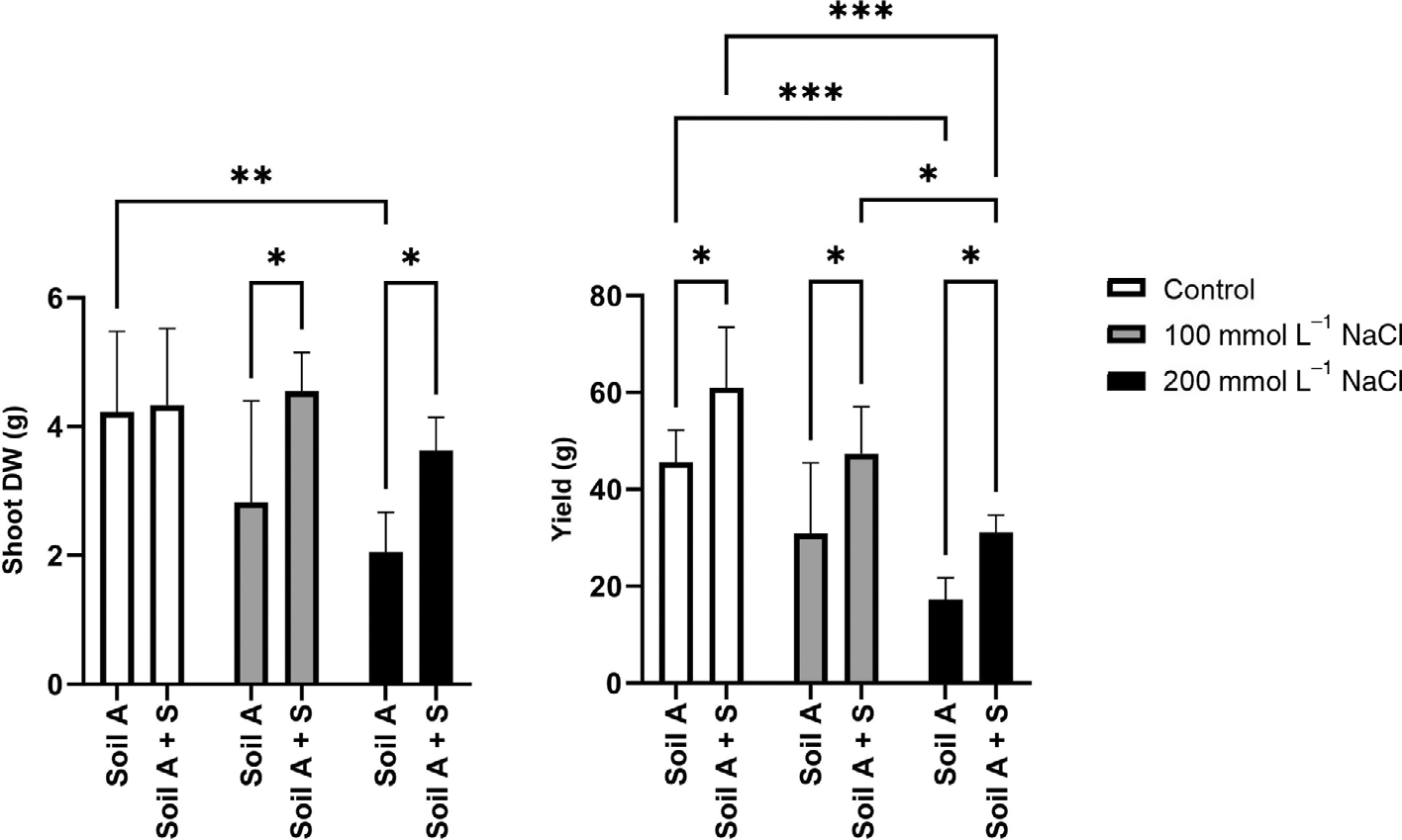
Shoot dry weight (DW) and yield of Tetragonia at Hf. Values are means (*n* = 6) ± standard deviation. Asterisks denote statistically significant differences at *P* < 0.05 (two‐way ANOVA and Tukey's test).

While there were no significant differences in non‐saline conditions, in saline conditions shoot dry weight (DW) was significantly higher for plants grown in Soil A + S compared to plants grown in Soil A. This increase was of about 60% in 100 mmol L^−1^ NaCl‐treated plants and of approx. 90% in 200 mmol L^−1^ NaCl‐treated plants. When comparing the effect of soil type on yield (i.e., the fresh weight (FW) of the edible portion of the shoot) in salt‐treated plants, plants grown in Soil A consistently produced a significantly lower yield compared to plants grown in Soil A + S. Indeed, compared to plants grown in Soil A, the yields obtained in Soil A + S were approximately 50% higher with 100 mmol L^−1^ NaCl and 80% higher when irrigated with 200 mmol L^−1^ NaCl. A similar increase in yield was also visible in non‐saline conditions, with plants grown in Soil A + S characterized by a significantly higher yield. Given the difference in DW and FW observed under control conditions, this variation indicates changes in shoot water content. Furthermore, in both soil types, while fresh shoot biomass remained comparable to controls with 100 mmol L^−1^ NaCl, it significantly decreased in 200 mmol L^−1^ NaCl‐treated plants.

Fig 4 reports the leaf area cover of plants grown in the two tested soil types and at the three salinity treatments. Leaf area cover reflects plant biomass production, and, as previously observed for shoot DW and plant yield (Fig. 3), plants grown on both soils showed significantly lower values of leaf area cover in 200 mmol L^−1^ NaCl treatment compared to both 100 mmol L^−1^ NaCl‐treated plants and controls. Such decrease was of ~50% for plants grown on Soil A and of 38% for Soil A + S‐grown plants. Yield and leaf area cover exhibited a linear relationship (*R* ^2^ = 0.95, *P* < 0.001); thus, the second parameter being non‐destructive, it can be integrated to evaluate the plants' response to salinity over time.

**Figure 4:**
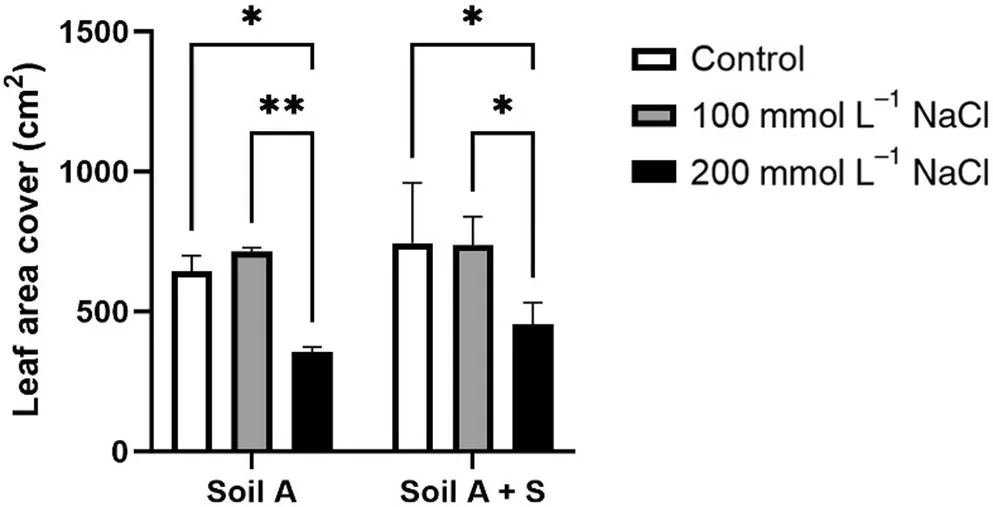
Leaf area cover at Hf. Values are means (*n* = 6) ± standard deviation. Asterisks denote statistically significant differences at *P* < 0.05 (two‐way ANOVA and Tukey's test).

### Water use

Fig 5 reports the plants' WUE and WP calculated at the end of the experiment. While no significant differences were seen for WUE, WP increased in 100 mmol L^−1^ NaCl‐treated plants compared to controls, for both soil types. Compared to controls, irrigating the plants with 100 mmol L^−1^ NaCl led to a 25% and 30% increase in WP in plants grown in Soil A and Soil A + S, respectively. The higher water consumption in controls compared to salt‐treated plants was further confirmed by the data shown in Fig. 6, which shows the pot weights for the control and 200 mmol L^−1^ NaCl treatments. With the peaks corresponding to the irrigation events, the differential slopes between two peaks under control and saline conditions indicate a higher water loss in control pots.

**Figure 5:**
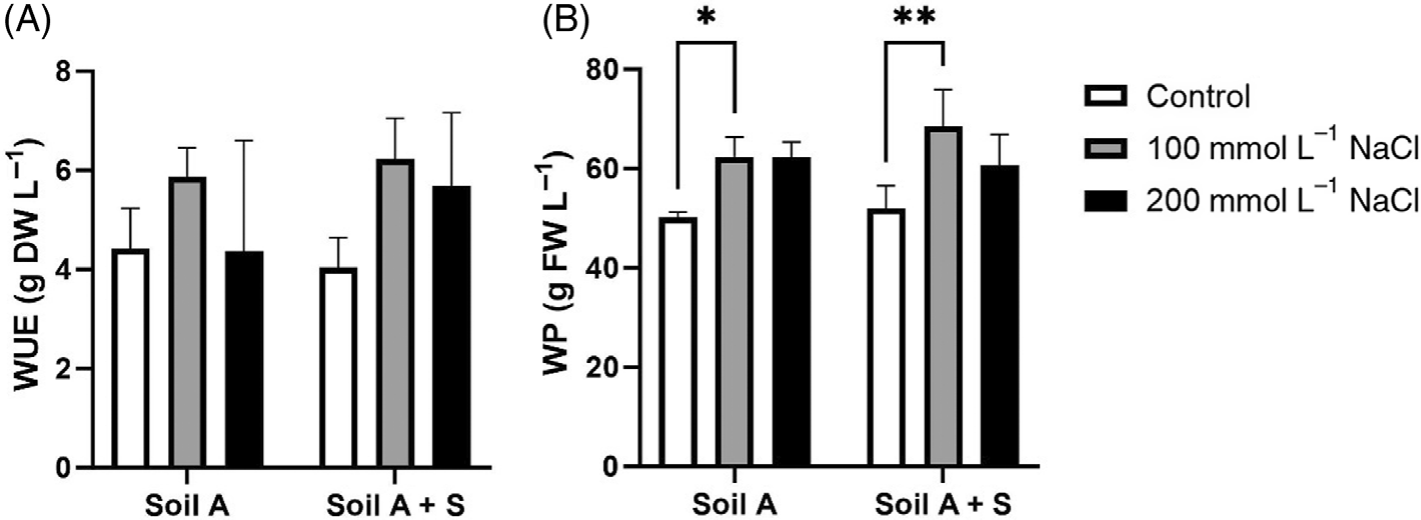
Plants' water use efficiency (A) and water productivity (B) at Hf. Values are means (*n* = 6) ± standard deviation. Asterisks denote statistically significant differences at *P* < 0.05 (two‐way ANOVA and Tukey's test). WUE, water use efficiency; WP, water productivity; DW, dry weight; FW, fresh weight.

**Figure 6:**
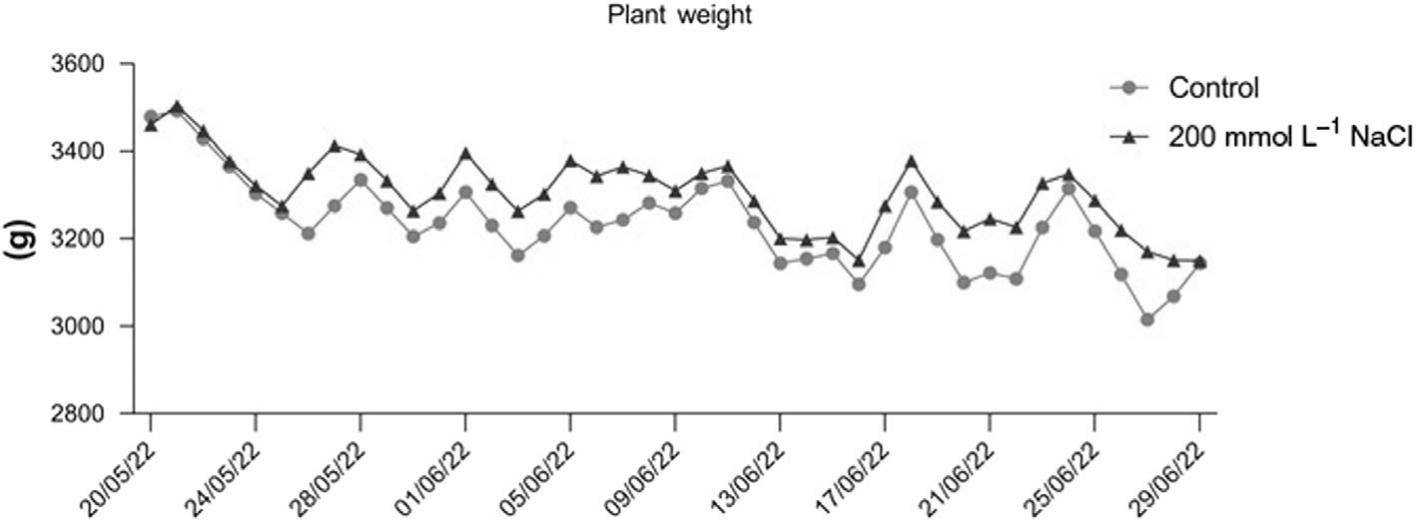
Pot weights collected from the logged scales. Values are means of the daily‐collected data.

### Leaf gas‐exchange results

As shown in Fig. 7, plants grown on Soil A + S had significantly higher stomatal conductance at T2 compared to Soil A‐grown plants. Nevertheless, before and after that time point, no other differences between plants grown in Soil A and Soil A + S emerged for either salinity treatment.

**Figure 7:**
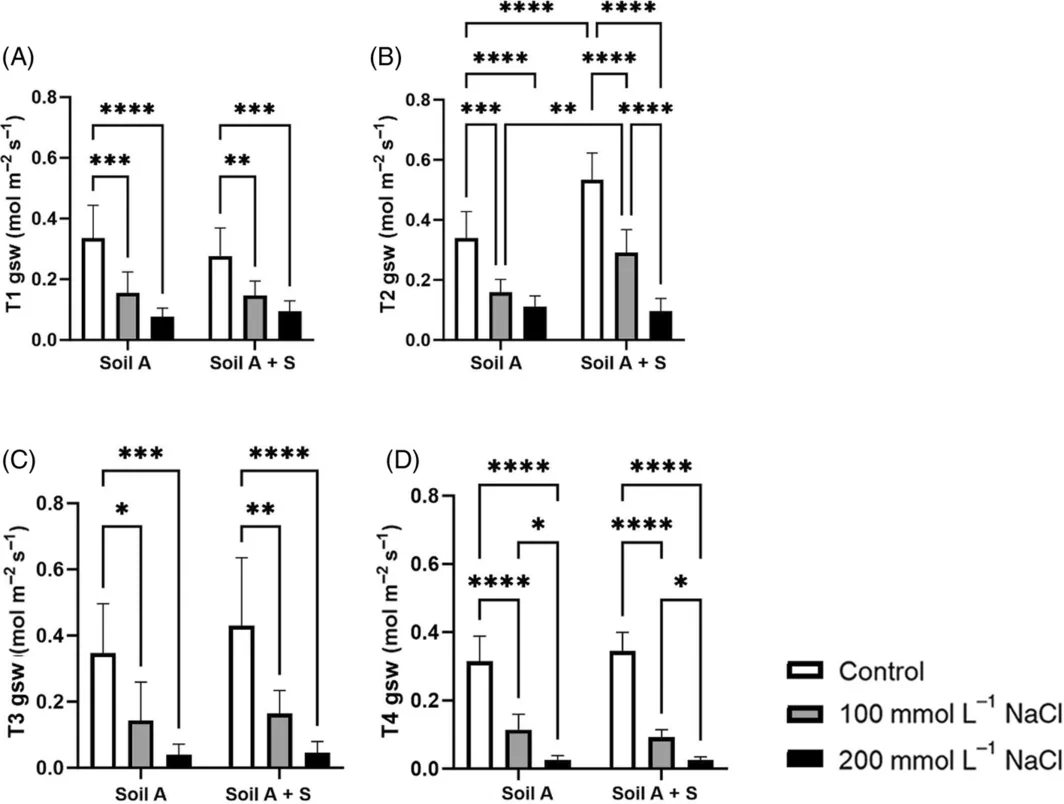
Stomatal conductance (gsw) at one, two (= Hi), three and four (= Hf) weeks after treatment introduction. Values are means (*n* = 6) ± standard deviation. Asterisks denote statistically significant differences at *P* < 0.05 (two‐way ANOVA and Tukey's test).

Fig 8 reports the results on the photosynthetic rate of plants at Hi and Hf. Prior to the start of the treatments, plants had similar photosynthetic activity across soil types, with values of 14.6 ± 4.8 for Soil A and 15.4 ± 1 for Soil A + S. The salt treatments, however, differentially affected plants in the two soil types. As observed for growth and leaf area data, highlighting significant differences between controls and 200 mmol L^−1^ NaCl treatments led to greater decline in assimilation rates compared with the 100 mmol L^−1^ NaCl treatment, with differences already evident at Hi for Soil A + S, while in Soil A they became significant only 2 weeks later at Hf.

**Figure 8:**
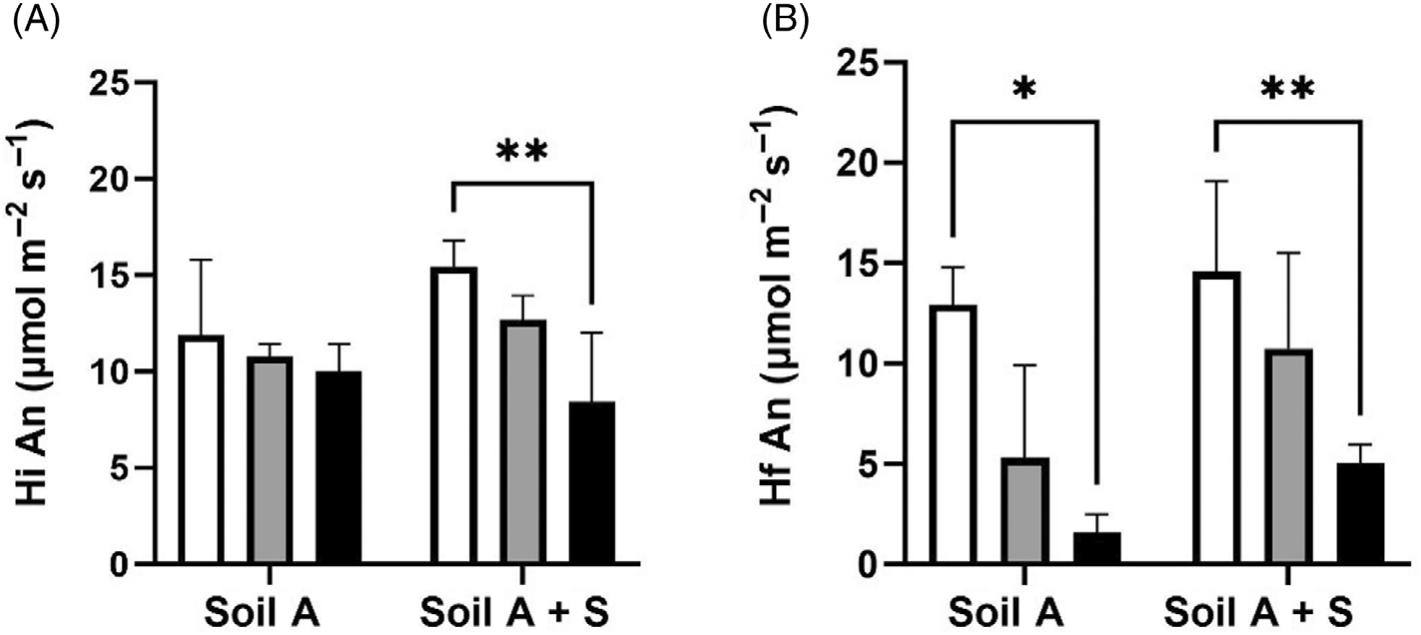
Photosynthetic rate (An) of plants at Hi (A) and Hf (B). Values are means (*n* = 6) ± standard deviation. Asterisks denote statistically significant differences at *P* < 0.05 (two‐way ANOVA and Tukey's test).

On the other hand, PSII quantum yields, collected on a weekly basis, did not show significant differences among salt treatments or soil types (Fig. 9).

**Figure 9:**
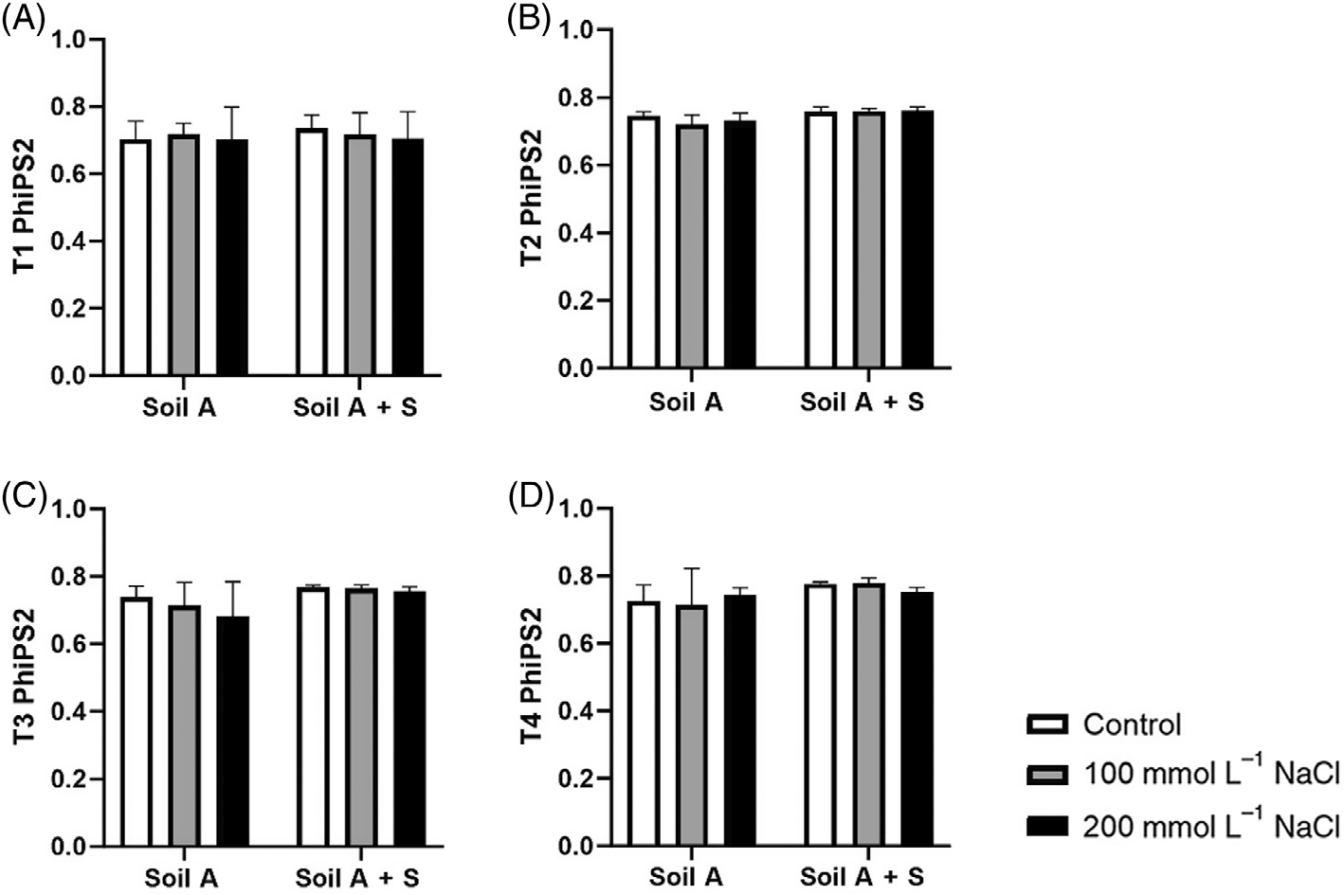
Actual quantum efficiency of photosynthetic electron transport through photosystem II at one week (A), two weeks (= Hi) (B), three weeks (C) and four weeks (= Hf) (D) after treatment introduction. Values are means (*n* = 6) ± standard deviation. Asterisks denote statistically significant differences at *P* < 0.05 (two‐way ANOVA and Tukey's test).

### Ion concentrations in edible leaves

Leaf Na and Cl concentrations increased significantly with increasing soil salinity, though the magnitude of the increments differed between the two ions and soil types (Fig. 10). Hence, independently of the salt treatment, leaf Na in salt‐treated plants doubled compared to controls, with no significant differences between salt treatments across the two soil types. By contrast, leaf Cl increased with soil salinity, showing significant increments between the 100 and 200 mmol L^−1^ NaCl treatments, with much larger increases in plants grown in Soil A than in those grown in Soil A + S. For example, in Soil A, leaf Cl in plants treated with 200 mmol L^−1^ NaCl was about twofold higher than that in plants grown with 100 mmol L^−1^ NaCl. By contrast, in Soil A + S, Cl levels at 200 mmol L^−1^ NaCl were only 1.2‐fold higher than at 100 mmol L^−1^ NaCl.

**Figure 10:**
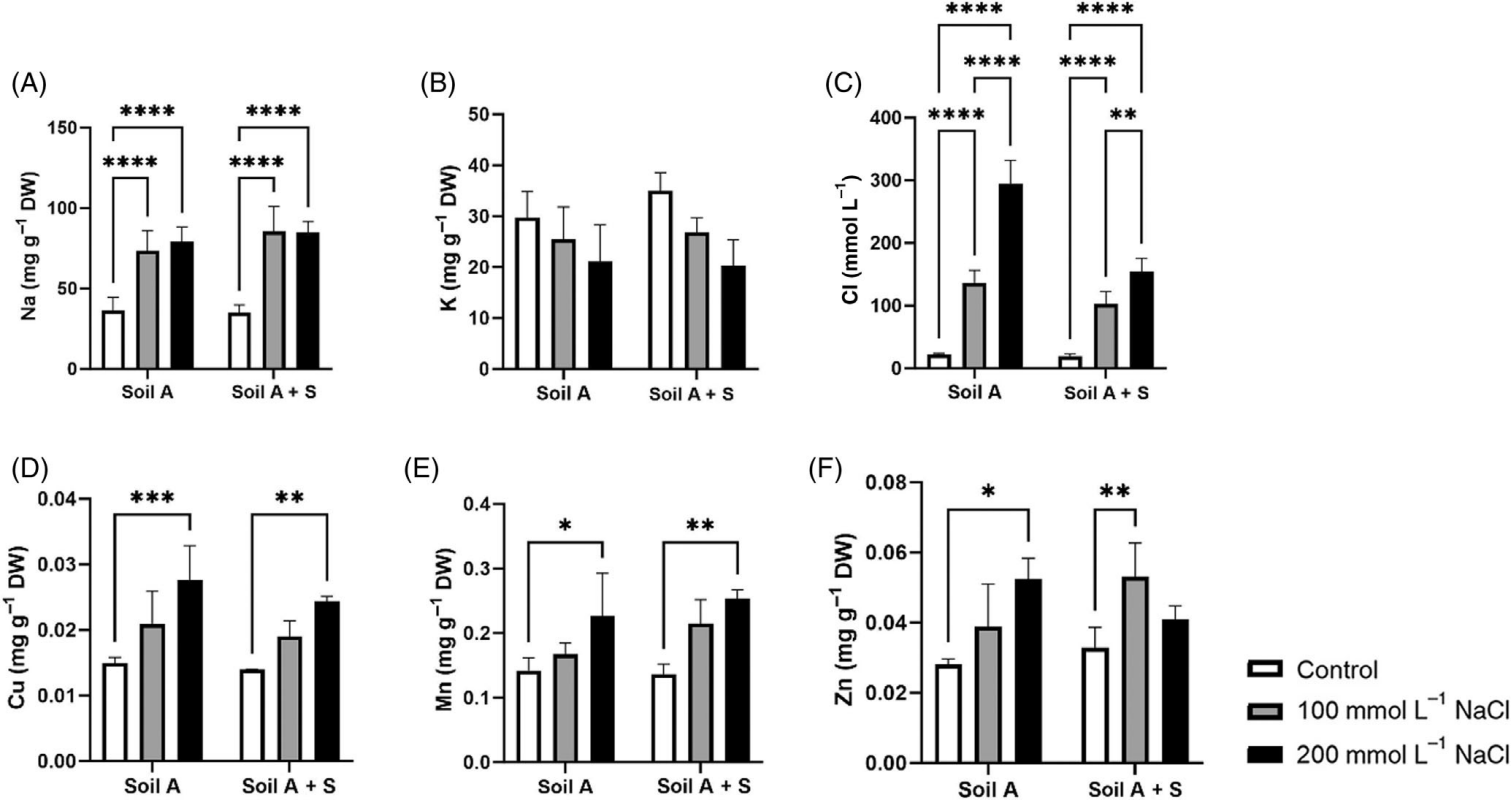
Ion concentration in leaves collected at Hf. Values are means (*n* = 6) ± standard deviation. Asterisks denote statistically significant differences at *P* < 0.05 (two‐way ANOVA and Tukey's test). DW, dry weight.

In contrast, with leaf Na and Cl, K concentrations declined, although not significantly, with increasing soil salinity across the two soil types. Interestingly, leaf Cu, Mn and Zn also increased in salt‐treated plants compared to controls.

## DISCUSSION

Tetragonia is a halophytic species whose growth performance was not inhibited by saline irrigation up to 100 mmol L^−1^ NaCl. By contrast, 200 mmol L^−1^ NaCl negatively affected all measured growth parameters (i.e., shoot DW and FW, leaf area cover). These results are only partially consistent with previous hydroponic studies, where 200 mmol L^−1^ NaCl negatively affected plant growth.^19^, ^21^, ^22^ However, while these previous studies reported stimulated growth and biomass production with 100 mmol L^−1^ NaCl compared to controls, this improved growth with 100 mmol L^−1^ NaCl did not occur in our soil‐based experiment. This discrepancy can be explained by salt accumulation processes occurring in the root zone under soil conditions, which do not occur in hydroponics thanks to the regular renewal of nutrient solution.^28^ Indeed, although the irrigation water contained only 100 mmol L^−1^ NaCl, soil EC values close to the roots exceeded 20 dS m^−1^ at the end of the experimental period, thus placing soil EC well beyond the optimal salinity for this halophyte. These findings highlight the importance of considering the soil matrix when evaluating plant salt tolerance, as the threshold determined in solution culture may not capture all key processes that might alter soil and water dynamics in soil conditions, thus affecting plant growth.^12^, ^23^

A key outcome of this study is the interaction between salinity and soil type. Under non‐saline conditions, the two soils supported similar biomass production. In contrast, under saline irrigation, plants grown in Soil A + S consistently outperformed those grown in Soil A. This suggests that soil physical properties, particularly texture and porosity, significantly modulated plant response to salinity. Indeed, post‐harvest analyses showed that EC in Soil A reached values up to 2.2‐fold higher than those in Soil A + S under 200 mmol L^−1^ NaCl. These differences, despite the same irrigation and nutrient regime, indicate that Soil A retained more salts in the root zone, while the sandier texture of Soil A + S enhanced leaching and reduced salt accumulation. Similar interactions between soil structure, salt retention and plant growth under saline irrigation have been reported in other halophytes and crop species.^29^, ^30^

Differences in salt distribution between soils were also reflected in leaf ion accumulation patterns. Leaf Cl was significantly lower in plants grown on Soil A + S, while Na values were comparable across soils. This suggests that Tetragonia may have a limited capacity for Na storage, as leaf Na appeared to reach a saturation point already at 100 mmol L^−1^ NaCl. Na is described as a non‐essential but beneficial (up to certain levels) nutrient, especially for halophytes,^31^, ^32^, ^33^ and more recently Cl has been described as a beneficial macronutrient, improving water balance and plant productivity.^34^, ^35^ Knowing the maximum sodium concentration achievable per unit weight of leaf tissue could be relevant for phytodesalination projects aimed at growing Tetragonia to restore salinized land, whether alone or intercropped with a salt‐sensitive crop. Indeed, both the increasing Na concentration and the observed reduction in soil EC close to the root collar in both soil types when irrigated with saline waters indicate an active removal of salts from the rhizosphere. These results align with previous studies on Tetragonia in hydroponics and soilless systems,^19^, ^22^ supporting the potential of Tetragonia for phytodesalination purposes and confirming its suitability for biosaline agriculture.

Under saline conditions, an increase in Na and a decrease in K is often observed, with the accumulation of Na coming at the cost of K and resulting in high Na/K ratios. However, a cytosolic Na/K ratio smaller than 1 does not seem generally essential for halophytes tolerance to high salinity conditions.^36^ Similarly, in the present experiment, we observed a declining trend in K concentration with increasing salinity, probably due to Na/K competition. Nevertheless, such a decrease was not significant, and K concentration never dropped below the deficiency threshold (i.e., below 10 mg g^−1^ DW (or 1%)), even at the highest salinity tested (with leaf K being ~20 mg g^−1^ DW in 200 mmol L^−1^ NaCl‐treated plants).

Focusing specifically on the physiological level, the fact that PSII efficiency remained stable across treatments suggests that the observed reduction in stomatal conductance under salinity likely contributed to photosynthetic downregulation.^37^ Moreover, the increase in WP observed in salt‐treated plants is most likely also related to the transpiration limitation. These results are consistent with previous studies reporting enhanced photoprotective responses and efficient reactive oxygen species scavenging in halophytes under saline stress.^38^

Salinity also affected mineral nutrition, leading to higher leaf concentrations of micronutrients such as Cu, Mn and Zn in salt‐treated plants compared to controls. Similar trends have been observed in Tetragonia and in other edible halophytes (i.e., *Mesembryanthemum crystallinum, Tetragonia decumbens*, *Salsola soda*).^39^, ^40^, ^41^ Interestingly, as mentioned above, leaf K concentration remained above the deficiency threshold, indicating that Tetragonia can maintain adequate K nutrition under stress conditions. The above‐described mineral enrichment is particularly important because, while mineral nutrients are essential to crops and can protect plants against both abiotic and biotic stresses,^42^ the same nutrients are also important in human nutrition. In particular, of the (at least) 49 nutrients required by humans to meet their metabolic needs, 22 are mineral elements, and the diet of over two‐thirds of the world's population lacks one or more essential mineral elements, with inadequate consumption resulting in adverse metabolic disturbances.^43^, ^44^ The reported nutrient enrichment thus has dual significance as it contributes to plant salt tolerance and enhances the nutritional value of edible biomass. Nevertheless, the concomitant accumulation of Na, Cl and potentially oxalates must be carefully considered for food use.^45^, ^46^

Overall, the integration of plant responses with post‐harvest soil analyses provides a mechanistic explanation for the observed effects of salinity and soil type. Soil A + S, due to its higher porosity, allowed greater leaching and lower salt accumulation, resulting in better plant performance under saline irrigation. These results underline the necessity of assessing both plant traits and soil properties when evaluating halophyte crops for saline agriculture. In this context, Tetragonia emerges as a promising crop not only for its yield stability under salinity but also for its potential role in phytodesalination and mineral enrichment of edible tissues. Future research should further explore its long‐term effects on soil properties and the balance between beneficial and antinutritional compounds in edible biomass.

## CONCLUSIONS

Salinity limits agricultural productivity globally, particularly in arid and semi‐arid regions where soil salinization is prevalent. Our results show that *Tetragonia tetragonioides* can be successfully cultivated under saline conditions, maintaining growth up to 100 mM NaCl and effectively taking up salts from the growing medium, highlighting its potential for saline agriculture and phytodesalination. Moreover, its mineral accumulation patterns highlight potential applications in biofortification strategies. Nevertheless, this study was conducted under controlled conditions and with a limited salinity range, without assessing field‐scale performance. Future research should therefore evaluate Tetragonia in open‐field trials across diverse pedoclimatic contexts to quantify its contribution to soil desalination on larger scales and explore its integration into diversified cropping systems.

## FUNDING INFORMATION

This project is part of the ERA‐NET Co‐fund FOSC, which has received funding from the European Union's Horizon 2020 research and innovation program, under grant agreement No. 862555.

## Data Availability

The data that support the findings of this study are available from the corresponding author upon reasonable request.
